# The influence of class mobility on pain empathy among the wealthy in collectivist cultures: evidence from ERPs

**DOI:** 10.3389/fnhum.2025.1675144

**Published:** 2026-01-21

**Authors:** Bingni Huang, Jinwen He, Xiaomin Wu, Jiaxian Luo, Yanshan Zhang, Pinchao Luo

**Affiliations:** 1 School of Psychology, South China Normal University, Guangzhou, China; 2 Department of Public Health, Shenzhen Clinical Research Center for Mental Disorders, Shenzhen Mental Health Center, Shenzhen Kangning Hospital, Shenzhen, China; 3 Key Laboratory of Brain, Cognition and Education Sciences, Ministry of Education, Guangzhou, China; 4 Center for Studies of Psychological Application, South China Normal University, Guangzhou, China; 5 Guangdong Key Laboratory of Mental Health and Cognitive Science, South China Normal University, Guangzhou, China

**Keywords:** class mobility, collectivist culture, event-related potentials (ERPs), LPP, N2, pain empathy

## Abstract

**Introduction:**

As a privileged group within the social structure, the wealthy play a significant role in social philanthropy and public welfare initiatives, making their empathic capacity a subject of considerable interest. Previous research has found that wealthy individuals who have experienced upward class mobility paradoxically demonstrate reduced empathy toward the poor. However, these studies were predominantly conducted in individualistic cultural contexts, leaving the collectivist cultural perspective largely unexplored.

**Methods:**

The present study employed a virtual-society paradigm to experimentally simulate class mobility in a collectivist context. Objective mobility direction (upward vs. horizontal) and subjective evaluation of mobility (positive vs. negative) were manipulated to construct four types of wealthy roles (upward-positive, upward-negative, horizontal-positive, horizontal-negative). Chinese university students from a collectivist cultural background were instructed to sequentially adopt each wealthy role and judge painful versus neutral pictures of the same low-status target, while event-related potentials (ERPs) were recorded.

**Results:**

The results revealed that in the early N2 component, under upward mobility conditions, pain stimuli elicited significantly smaller N2 amplitudes compared to neutral stimuli, while no significant difference was observed under horizontal mobility conditions. In positive evaluation conditions, pain stimuli evoked significantly smaller N2 amplitudes than neutral stimuli, whereas no significant difference emerged in negative evaluation conditions. For the late LPP component, pain stimuli consistently elicited significantly larger LPP amplitudes than neutral stimuli, regardless of either the objective direction of class mobility or subjective evaluation.

**Discussion:**

These findings suggest that, within this simulated class-mobility context, upward mobility experiences and positive appraisal primarily influence early neural processing of the poor target’s pain, while later evaluative processing remains relatively stable. This study provides neural-level evidence for understanding how class mobility affects pain empathy among the wealthy in collectivist cultures, thereby enriching research on the relationship between social stratification and pain empathy.

## 1 Introduction

Empathy refers to an individual’s capacity to share and understand the emotions and feelings of others, which forms the foundation for deep interpersonal connections and mutual understanding (Sprong et al., 2019). Empirical research extensively demonstrates that empathy promotes prosocial behavior, fostering cooperation, mutual assistance, and social cohesion (Acevedo et al., 2012). By empathizing with others, individuals from different social strata can overcome stereotypes and prejudice, enhance mutual understanding and communication, and become more willing to help and support one another (Decety and Jackson, 2006), thereby contributing to social harmony.

As a privileged group within the social structure, the wealthy play a significant role in social philanthropy and public welfare initiatives, which makes their empathic capacity a subject of considerable interest. With their substantial economic, social, and cultural capital, they are well-positioned to support societal stability and development through charitable donations, public welfare investments, and social welfare programs (Kraus et al., 2012). Due to their abundant resources, society holds high expectations for the wealthy to engage in prosocial behaviors, assist disadvantaged groups, and contribute to societal well-being (Koo et al., 2023). As one of the key drivers of prosocial behavior, empathy has thus become a focal psychological capacity for the wealthy (Acevedo et al., 2012).

The wealthy can be categorized based on how they acquired their wealth. One group comprises individuals who have achieved upward class mobility, rising from lower-middle-class backgrounds or middle-class backgrounds through personal effort; the other includes those born into affluence who have remained within their inherited class status, experiencing horizontal mobility (Leckelt et al., 2022; Hansen, 2014). Public perception generally assumes that those who have experienced upward mobility, having endured poverty or lower-class living conditions, should demonstrate stronger empathic responses (Batson et al., 1996; Hodges et al., 2010), more favorable attitudes toward the poor (Ruttan et al., 2015), and greater support for social welfare policies (Koo et al., 2023). In contrast, individuals born wealthy, who lack direct exposure to poverty, may exhibit certain deficits or barriers in empathic capacity (Leckelt et al., 2022).

However, current research reveals two counterintuitive findings. First, compared to lower-income individuals, the wealthy tend to exhibit higher egocentricity and face greater challenges in empathic capacity (Piff et al., 2010; Koo et al., 2023), whereas the poor demonstrate stronger outward orientation and heightened empathic responses (Kraus et al., 2012). Second, experimental studies show that when participants imagine experiencing upward social mobility from lower to higher class status, they systematically underestimate the difficulties of such mobility. These individuals are more likely to attribute poverty to personal lack of effort rather than structural factors, consequently showing reduced empathy toward the struggles of lower-class individuals (Koo et al., 2023). Notably, these findings primarily emerge from individualistic cultural contexts where personal achievement and independence are highly valued (Triandis and Gelfand, 2012; Santos et al., 2017). This cultural framework may weaken emotional connections and shared identity across class boundaries while reinforcing the wealthy’s identification with their current privileged status. Simultaneously, it increases psychological distance from disadvantaged groups (e.g., low-income populations), ultimately resulting in diminished interclass empathy.

The mechanisms of empathy exhibit significant differences between individualistic and collectivistic cultural contexts. In individualistic cultures, empathy is typically directed toward close relationships, such as family and friends, and heavily depends on personal autonomy and self-referential processing, resulting in weaker empathic responses toward strangers or out-group members (Piff et al., 2010). For instance, among Western participants observing others in pain, activation of the anterior insula is strongly associated with self-referential emotional processing, suggesting that empathic responses rely on personal emotional resonance (Decety and Jackson, 2006).

In contrast, collectivist cultures emphasize group harmony, interdependence, and sensitivity to others’ needs (Brewer and Chen, 2007), fostering socially oriented empathy that is highly responsive to contextual and normative cues (Duan et al., 2008). For example, individuals from collectivist societies such as China and Japan often actively adjust their behavior to align with prevailing social norms when witnessing others’ suffering (Yang et al., 2024; Klein et al., 2024). An intriguing question arises: If the impact of class mobility experiences on the empathy of the wealthy is examined within a collectivistic cultural framework, would public expectations be met as anticipated?

Social Mobility Theory posits that individuals may experience shifts in their social class positions, encompassing both objective mobility (upward, horizontal, or downward) and subjective evaluations of these mobility experiences (positive or negative) (Koo et al., 2023; van Dijk et al., 2015). Drawing on this framework, the present study employed a virtual society paradigm and ERP technology to examine empathy in college students from a collectivist cultural background in China. Based on the objective direction (upward vs. horizontal) and subjective evaluation (positive vs. negative) of class mobility, participants were instructed to adopt four types of wealthy roles: upward-positive, upward-negative, horizontal-positive, and horizontal-negative types. They were instructed to adopt these assigned roles while viewing pain and neutral images of impoverished individuals, and their neural responses were recorded using ERP methodology. This design allows for a fine-grained examination of how simulated class mobility experiences shape neural indices of pain empathy among the wealthy roles within collectivist cultures, thereby enriching research on the relationship between social stratification and pain empathy.

We hypothesized that collectivist cultural norms may buffer the negative effects of class mobility on empathy: (1) wealthy individuals who have experienced upward class mobility may prioritize social connectedness and exhibit stronger empathic responses when observing the suffering of the poor; (2) compared to negative evaluations, those with positive evaluations of their mobility experience may view their upward mobility as “hard-earned success.” This perspective facilitates greater empathy toward the poor, as it leads to “moral self-expansion” (Aquino and Reed, 2002; Reed and Aquino, 2003), where social norms and others’ needs are incorporated into one’s self-concept; and (3) both the objective direction and subjective evaluation of class mobility may independently or interactively influence neural responses of pain empathy among the wealthy.

## 2 Materials and methods

### 2.1 Participants

The required sample size was estimated using G*Power 3.1. With an alpha level of 0.05 and an effect size of 0.25, a minimum of 38 participants was needed to achieve 95% statistical power. A total of 44 university students were recruited for the study. Five participants were excluded due to artifact segments exceeding 10% of total trials during preprocessing, resulting in 39 valid participants (15 males, 24 females; mean age = 20.77 ± 1.61 years). All participants were right-handed, had normal or corrected-to-normal vision, and reported no history of neurological, brain injury, or developmental disorders. Participants’ family SES scores were centered around the sample mean with limited variability, suggesting a relatively homogeneous university sample without pronounced extremes of affluence or deprivation. Written informed consent was obtained from all participants prior to the experiment, and they received monetary compensation upon completion. The study protocol was approved by the Ethics Committee of South China Normal University and adhered to the international research principles involving human participants outlined in the Declaration of Helsinki (World Medical Organization, 1999).

### 2.2 Family socioeconomic status

Objective family socioeconomic status (SES) was assessed with the Family Socioeconomic Status Questionnaire developed by Shi and Shen (2007). The scale includes five indicators: father’s and mother’s occupational status, father’s and mother’s educational attainment, and monthly household income. All five indicators were standardized and combined using factor analysis to yield a composite family SES score (Wang et al., 2016), with higher scores indicating higher objective socioeconomic status.

### 2.3 Stimuli

The pain stimuli consisted of photographs depicting body parts (hands, forearms, feet) in painful or neutral situations, which have been widely used in previous research (Fan and Han, 2008; Decety et al., 2010; Luo et al., 2018a). Each neutral stimulus corresponded to a pain image but without painful elements. A total of 40 pain and 40 neutral images were used.

### 2.4 Experimental procedure

The experiment was conducted in a quiet, dimly lit, sound-attenuated room. The experimental procedure was programmed using E-Prime 2.0 and presented on a 17-inch color monitor. Upon arrival, participants were first briefed by the experimenter about the experimental procedure and important instructions. After understanding the process and instructions, participants were asked to read and sign the informed consent form. Participants then completed questionnaires, including a Family SES questionnaire and demographic information.

Following previous studies employing virtual society paradigms (Sprong et al., 2019; Du et al., 2021; Koo et al., 2023), participants were required to read one of four background profiles of characters living in a fictional society named “Jijing.” Each profile detailed the individual’s social mobility history and current socio-economic status, helping participants understand their assigned roles. Specifically, participants were asked to adopt four different wealthy personas:

Upward-positive types: Huoying was born into an extremely poor farming family. He studied diligently and worked hard from a young age. He was admitted to a prestigious university and, after graduation, was recruited through campus placement by a large state-owned enterprise. After working there for 6 years, he built a strong network and accumulated some wealth. He then left the company to start his own business. After 10 years of hard work, he is now the CEO of a publicly listed company. He feels proud of his past experiences and often talks about them in public.

Upward-negative types: Lijia was born into an extremely poor farming family. He studied diligently and worked hard from a young age. He was admitted to a prestigious university and, after graduation, was recruited through campus placement by a large state-owned enterprise. After working there for 6 years, he built a strong network and accumulated some wealth. He then left the company to start his own business. After 10 years of hard work, he is now the CEO of a publicly listed company. However, he fears being ridiculed for his humble background and often avoids mentioning his past.

Horizontal-positive types: Lize was born into a wealthy family. With the advantages brought by his family background, he enjoyed the best educational opportunities from an early age. After graduating from a prestigious university abroad, he returned to China and joined the family business. He succeeded his father as the CEO of the family’s publicly listed company. He feels proud of his past experiences and often talks about them in public.

Horizontal-negative types: Xianghua was born into a wealthy family. With the advantages brought by his family background, he enjoyed the best educational opportunities from an early age. After graduating from a prestigious university abroad, he returned to China and joined the family business. He succeeded his father as the CEO of the family’s publicly listed company. However, he fears being ridiculed as a privileged second-generation heir and often avoids mentioning his past.

To minimize the potential impact of participants’ varying socioeconomic backgrounds, a within-subjects design and ABBA counterbalancing were employed to randomize the order of role presentation, reducing variability and minimizing potential biases or fatigue effects. Additionally, to allow participants to switch roles smoothly, they were assigned to one role per week, rotating through all four wealthy character roles across 4 weeks, completed the experiment while adopting each role.

Before each weekly experiment, participants first viewed the corresponding character profile. The experimenter then guided them to verbally recall their life experiences in “Jijing” and reflect on their self-evaluation of those experiences, helping to strengthen their role engagement. To further ensure role immersion, before the EEG experiment began participants answered a yes/no screening question about whether they felt sufficiently immersed in the assigned role. Only participants who responded “yes” to this question were allowed to proceed to the next phase of the experiment. Subsequently, participants rated the current socioeconomic status, pre-university socioeconomic status, and perceived social mobility of the assigned role using 9-point Likert scales (higher scores indicating higher class or greater mobility).

Next, participants proceeded to the pain empathy task, which measured their empathy toward individuals from lower socioeconomic backgrounds. The experiment tasks helped participants maintain cognitive and emotional engagement with their roles through situational simulations and guided language. For example, while viewing images, participants were prompted to think about the social background and emotional reactions of the role they were playing, enhancing their immersion.

Participants were introduced to Wang Ming, a character living in “Jijing”:

Lower-class: Wang Ming was born into an ordinary family. Both of his parents were factory workers. He attended regular public schools throughout his childhood. After passing the college entrance examination, he was admitted to a second-tier university. After graduation, he joined’ a private company and started working from an entry-level position. Even after 10 years of work, he remains in a basic position and struggles to make ends meet.

**FIGURE 1:**
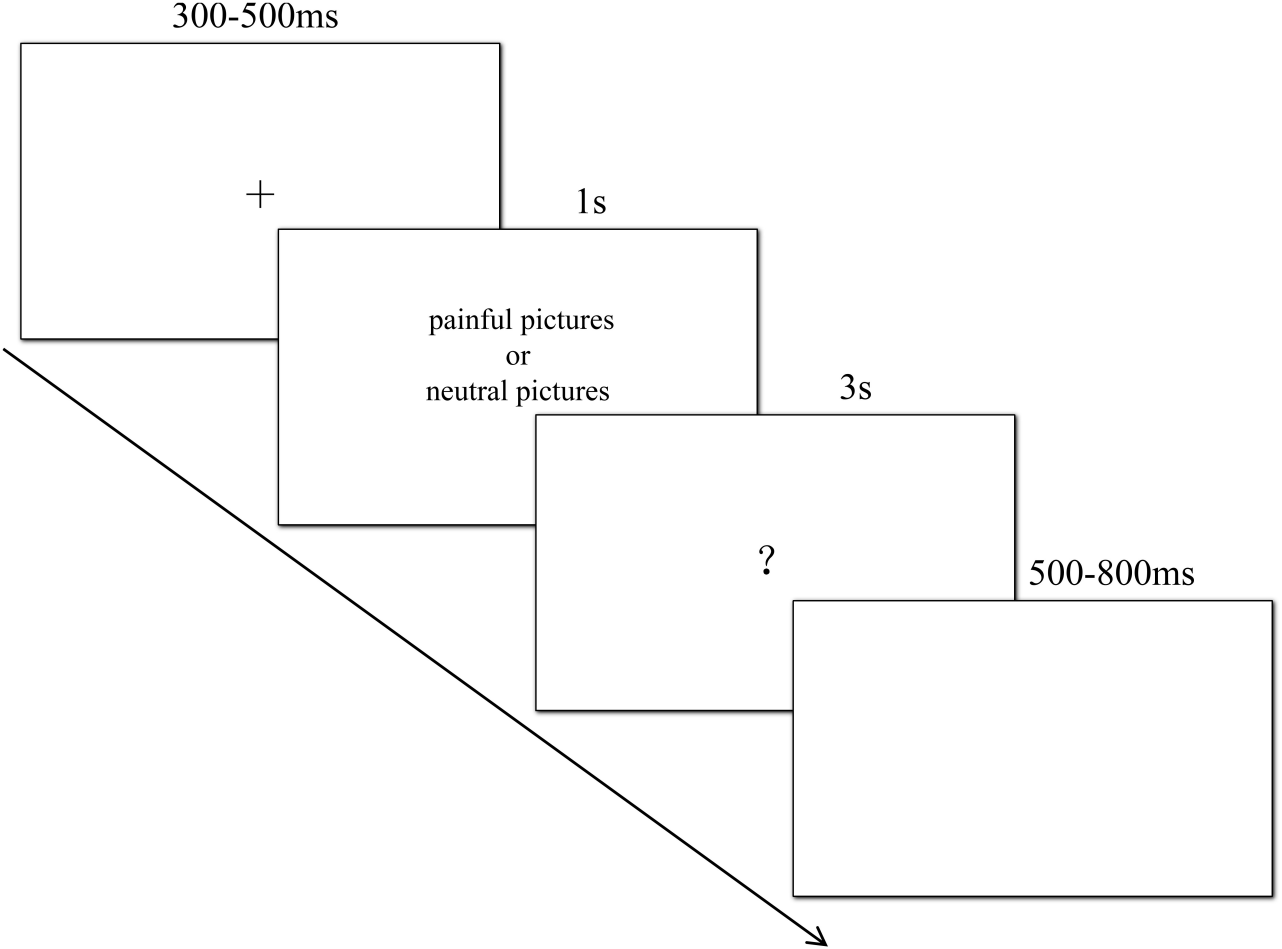
Procedure of a single trial.

After learning about Wang Mings background, participants were informed that they would next view images depicting Wang Ming’s bodily pain. Each trial began with a fixation cross (“+”) presented for 300–500 ms, followed by a pain or neutral image of Wang Ming’s body part displayed for 1000 ms. Next, a question mark appeared, prompting participants to quickly classify the image as painful or neutral via button press. Responses exceeding 3000 ms were recorded as omissions. A blank screen (500–800 ms) concluded each trial. The EEG task consisted of 4 blocks, each containing 80 trials with randomized presentation of pain and neutral images. The procedure of a single trial is illustrated in Figure 1.

After the EEG task, participants completed a behavioral assessment. Each block contained 20 trials. During each trial, after the image presentation, participants responded to two questions: (1) perceived pain intensity of Wang Ming, and (2) personal discomfort experienced when viewing the image. Both ratings were made on 9-point Likert scales (higher scores indicating stronger perceptions).

### 2.5 Data recording and processing

EEG data were recorded using a 64-channel cap (Brain Products, Germany) based on the extended International 10–20 system. The ground electrode was placed on the forehead, and FCz served as the reference during recording. EEG signals were filtered online with a bandpass of 0.01–100 Hz and sampled at 1000 Hz. Electrode impedances were maintained below 5 kΩ. Offline analysis re-referenced signals to the averaged mastoids and applied a 0.1–40 Hz bandpass filter with automatic artifact correction. The analysis epoch ranged from 200 ms before to 1000 ms after stimulus onset, with baseline correction using the 200 ms pre-stimulus interval. Trials containing artifacts exceeding ±80 μV (e.g., muscle or cardiac artifacts) were manually excluded. Ocular and movement artifacts were removed via independent component analysis (Jung et al., 2001). Across the four weekly sessions, all recordings used the same EEG system, the same experimenter, and a standardized electrode placement procedure, ensuring comparable electrode positioning and signal quality over time.

Following prior research (Fan et al., 2021; Luo et al., 2018a,b) and based on topographical and waveform inspection, nine electrodes were selected for statistical analysis: Fz, F3, F4 (frontal), Cz, C3, C4 (central), and Pz, P3, P4 (parietal). The fronto-central electrodes (Fz, F3, F4, Cz, C3, C4) were used to compute regional averages for the N2 component (200–300 ms), and the centro-parietal electrodes (Cz, C3, C4, Pz, P3, P4) were used for the LPP component (450–750 ms). Repeated-measures ANOVAs with 2 (mobility direction: upward, horizontal) × 2 (evaluation: positive, negative) × 2 (stimulus type: pain, neutral) factors were conducted on the mean amplitudes of N2 and LPP. Greenhouse-Geisser correction was applied to *p*-values and degrees of freedom when necessary.

## 3 Results

### 3.1 Manipulation check for class mobility

To verify the effectiveness of class mobility manipulation, we first calculated the difference scores between participants’ self-reported current socioeconomic status and their pre-university socioeconomic status under both upward and horizontal mobility conditions. Paired-sample *t*-tests revealed that the perceived class difference was significantly greater in the upward mobility condition (*M* = 7.00, SD = 1.33) than in the horizontal mobility condition (*M* = 0.11, SD = 1.79), t(38) = 13.46, *p* < 0.001. Additionally, perceived mobility scores were significantly higher in the upward mobility condition (*M* = 6.53, SD = 0.70) than in the horizontal mobility condition (*M* = 2.53, SD = 1.90), t(38) = 8.63, *p* < 0.001. These results confirm the successful manipulation of class mobility.

### 3.2 Manipulation check for empathic response

Following prior studies (Lamm et al., 2011; Sheng and Han, 2012), correlation analyses were conducted to ensure that participants’ responses reflected genuine empathic engagement rather than simple emotional evaluation. Significant positive correlations were found between participants’ ratings of Wang Ming’s perceived pain intensity and their self-reported discomfort for both pain and neutral images. For pain images, *r* = 0.76, *p* < 0.001; for neutral images, *r* = 0.90, *p* < 0.001. These findings indicate that the task successfully elicited empathic responses.

### 3.3 ERP results

**FIGURE 2:**
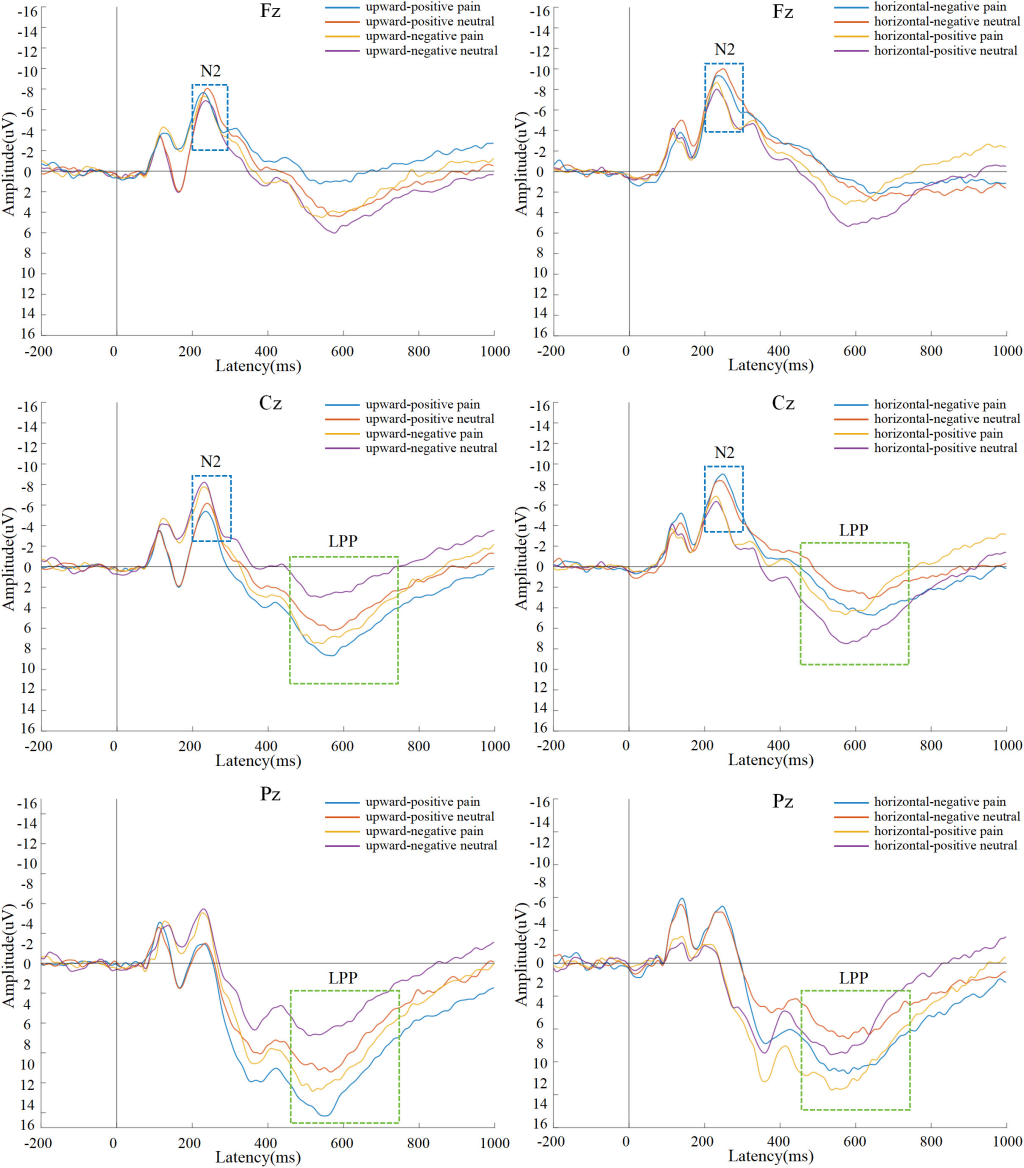
Grand-average waveforms at Fz, Cz, and Pz.

**FIGURE 3:**
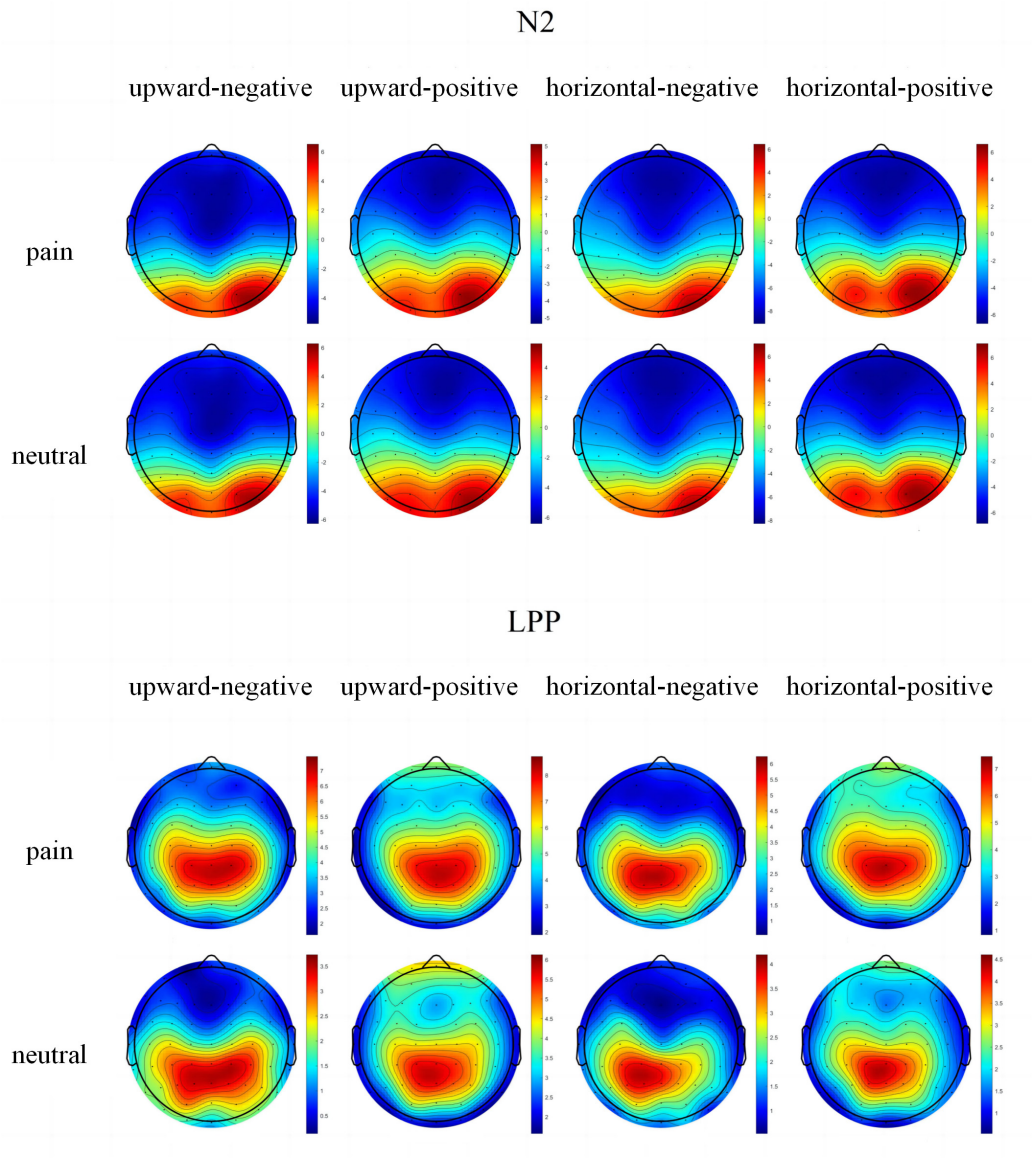
Topographical maps of N2 and LPP components under each condition.

After the electrical signal data recorded in the EEG experiment were averaged across trials, the waveform and topographic maps of ERP components under different conditions are shown in Figures 2, 3.

#### 3.3.1 N2 component

Descriptive statistics for mean N2 amplitudes across conditions are presented in Table 1.

**TABLE 1:** Descriptive statistics for N2 mean amplitudes.

Mobility direction	Evaluation	Pain stimuli (*M* ± SD)	Neutral stimuli (*M* ± SD)
Upward	Positive	−3.34 ± 3.58	−4.18 ± 2.94
	Negative	−4.32 ± 4.91	−4.79 ± 4.68
Horizontal	Positive	−4.33 ± 3.17	−4.59 ± 3.69
	Negative	−6.70 ± 4.47	−6.08 ± 4.62

A 2 (mobility direction: upward, horizontal) × 2 (evaluation: positive, negative) × 2 (stimulus type: pain, neutral) repeated-measures ANOVA was performed on mean N2 amplitudes. The main effects of mobility direction, *F*(1, 38) = 0.98, *p* = 0.329, η*_*p*_*^2^ = 0.03; evaluation, *F*(1, 38) = 1.13, *p* = 0.295, η*_*p*_*^2^ = 0.03; and stimulus type, *F*(1, 38) = 3.50, *p* = 0.070, η*_*p*_*^2^ = 0.09, were not significant.

A significant interaction between mobility direction and stimulus type was found, *F*(1, 38) = 10.52, *p* = 0.003, η*_*p*_*^2^ = 0.23. Simple effects analysis showed that under upward mobility, pain stimuli elicited significantly smaller N2 amplitudes (*M* = −3.78, SD = 4.14) than neutral stimuli (*M* = −4.45, SD = 3.72), *F*(1, 38) = 13.36, *p* < 0.001, η*_*p*_*^2^ = 0.28. Under horizontal mobility, no significant difference was found between pain (*M* = −5.45, SD = 3.92) and neutral stimuli (*M* = −5.30, SD = 4.10), *F*(1, 38) = 0.92, *p* = 0.343, η*_*p*_*^2^ = 0.03.

A significant interaction was also observed between evaluation and stimulus type, *F*(1, 38) = 5.85, *p* = 0.021, η*_*p*_*^2^ = 0.14. Under positive evaluation, pain stimuli elicited significantly smaller N2 amplitudes (*M* = −3.81, SD = 3.35) than neutral stimuli (*M* = −4.37, SD = 3.24), *F*(1, 38) = 9.94, *p* = 0.003, η*_*p*_*^2^ = 0.22. Under negative evaluation, no significant difference was found, *F*(1, 38) = 0.14, *p* = 0.711, η*_*p*_*^2^ = 0.004.

The interaction between mobility direction and evaluation was not significant, *F*(1, 38) = 0.19, *p* = 0.664, η*_*p*_*^2^ = 0.01, nor was the three-way interaction, *F*(1, 38) = 1.00, *p* = 0.324, η*_*p*_*^2^ = 0.03.

#### 3.3.2 LPP component

Descriptive statistics for mean LPP amplitudes across conditions are presented in Table 2.

**TABLE 2:** Descriptive statistics for LPP mean amplitudes.

Mobility direction	Evaluation	Pain stimuli (*M* ± SD)	Neutral stimuli (*M* ± SD)
Upward	Positive	6.68 ± 3.00	4.67 ± 2.51
	Negative	5.77 ± 3.11	2.71 ± 2.23
Horizontal	Positive	5.39 ± 2.26	3.04 ± 1.89
	Negative	4.36 ± 2.69	2.75 ± 2.64

A 2 (mobility direction: upward, horizontal) × 2 (evaluation: positive, negative) × 2 (stimulus type: pain, neutral) repeated-measures ANOVA was conducted on mean LPP amplitudes. A significant main effect of stimulus type was found, *F*(1, 38) = 70.02, *p* < 0.001, η*_*p*_*^2^ = 0.07. Pain stimuli (*M* = 5.55, SD = 0.45) elicited significantly larger LPP amplitudes than neutral stimuli (*M* = 3.29, SD = 0.38).

No significant main effects were found for mobility direction, *F*(1, 38) = 1.89, *p* = 0.178, η*_*p*_*^2^ = 0.05, or evaluation, *F*(1, 38) = 1.80, *p* = 0.188, η*_*p*_*^2^ = 0.05. Moreover, no significant interactions were observed between mobility direction and evaluation, *F*(1, 38) = 0.25, *p* = 0.623, η*_*p*_*^2^ = 0.01; mobility direction and stimulus type, *F*(1, 38) = 1.10, *p* = 0.301, η*_*p*_*^2^ = 0.03; or evaluation and stimulus type, *F*(1, 38) = 0.09, *p* = 0.770, η*_*p*_*^2^ = 0.002. The three-way interaction was also non-significant, *F*(1, 38) = 2.76, *p* = 0.106, η*_*p*_*^2^ = 0.07.

## 4 Discussion

This study examined how class mobility experiences influence pain empathy among wealthy individuals within a collectivist cultural context using a virtual society paradigm in which Chinese university students adopted wealthy roles with different objective mobility and subjective evaluations. The findings revealed that participants adopting an upwardly mobile wealthy role exhibited empathic responses starting from early automatic emotional sharing stages and continuing into later cognitive control stages. In contrast, those adopting horizontally mobile wealthy roles displayed empathic responses primarily during the later cognitive control stage. Moreover, regardless of mobility direction, positive evaluations of mobility experiences facilitated early-stage automatic emotional sharing.

At the early N2 stage, significant differences between responses to painful and neutral stimuli emerged only when participants adopted upwardly mobile wealthy roles or roles that positively evaluated their mobility history, when participants adopted horizontally mobile or negatively evaluating wealthy roles, this early pain-neutral difference was reduced or not significant. Moreover, at the later LPP stage, responses to painful stimuli were larger than responses to neutral stimuli across all conditions, and this effect was not modulated by either mobility direction or subjective evaluation. Overall, in this simulated class-mobility context, mobility experiences and their subjective appraisal appear to be primarily related to early processing of others’ pain, whereas later evaluative processing remains relatively stable across wealthy-role conditions.

Consistent with prior research, the N2 component serves as an early neural marker of automatic processing of pain stimuli and reflects the affective component of pain empathy (Chen et al., 2012; Cheng et al., 2012), and it may reflect a set of processes including automatic detection of others’ pain, affective arousal, habituation to pain cues, attentional control, and response conflict (Botvinick et al., 2004; Kerns et al., 2004; Luo et al., 2018a,b). In the present study, when participants adopted an upwardly mobile wealthy role, pain stimuli elicited significantly smaller N2 amplitudes compared to neutral stimuli, while no significant difference was observed when they adopted a horizontally mobile role.

First, from the perspective of automatic processing and habituation, smaller N2 amplitudes are typically taken to indicate that the target stimuli are processed in a more familiar and efficient manner, with reduced demands on vigilance and monitoring resources. This suggests that wealthy individuals who have experienced upward mobility may find it easier to process pain-related stimuli. One possible reason is that upwardly mobile individuals often start out in lower social classes, and the present paradigm also explicitly simulates their trajectory from a low to a high social position. Thus, during their development they are more likely to have encountered, understood, or even personally experienced the kinds of hardships faced by low-SES groups, such as resource scarcity, unsafe neighborhoods, unstable employment, and fluctuating educational opportunities (Kraus et al., 2012). After moving from a lower to a higher social class, when these individuals are confronted with low-status targets whose suffering resembles their own past experiences, the corresponding cues are both familiar and consistent with their autobiographical background, making them more sensitive to these familiar signals. As a result, in the early processing stage they can distinguish painful from neutral information using fewer resources and in a more fluent manner, which is reflected in reduced N2 amplitudes under the pain condition. In this sense, the simulated upwardly mobile wealthy individuals can process the poor target’s pain more easily and rapidly (Hein and Singer, 2008; Hodges et al., 2010).

Second, from the perspective of response conflict and conflict monitoring, the frontal-central N2 is also regarded as a neural response to incongruent or conflicting information in the task (Botvinick et al., 2004; Kerns et al., 2004). Larger N2 amplitudes are generally taken to reflect higher levels of conflict and inhibitory demands (Lamm et al., 2011). Compared with horizontally mobile wealthy individuals, those in the simulated upward-mobility condition may experience a stronger sense of role identification and self-other overlap with the low-status targets. Consequently, when they are faced with the suffering of people at the bottom of the social hierarchy, they may experience less internal conflict between “keeping distance and self-protection” and “approaching the other and empathizing.” In other words, this role identification not only enhances their motivation to empathize with the poor, but also reduces the need to suppress or avoid such emotional responses, thereby lowering the load on conflict monitoring and inhibition. This reduction in conflict-related processing is manifested as attenuated N2 amplitudes in the upward-mobility condition.

Additionally, this study found that only under positive evaluation conditions did pain stimuli evoke smaller N2 amplitudes than neutral stimuli. In temporal terms, the N2 primarily indexes an early, more automatic stage of stimulus processing, where discrimination relies more on stimulus-driven properties and less on additional top-down attentional control. Positive subjective evaluation is regarded as a relatively stable psychological resource that helps maintain a more balanced emotional state, enabling individuals to respond to external information in a calmer and more objective manner (Orth and Robins, 2014). Under such circumstances, when individuals in the positive-evaluation condition are confronted with both painful and neutral stimuli, early processing can focus more directly on the task of distinguishing stimulus types: pain cues, which are highly survival-relevant and threatening, have high discriminability and salience and can be rapidly extracted and processed by the brain without requiring substantial additional monitoring resources (Legrain et al., 2011; Torta et al., 2017), so the brain does not need to further amplify attentional engagement in order to detect and process them. By contrast, neutral stimuli that lack obvious threat require greater early attentional and monitoring resources to evaluate their meaning and task relevance, which is reflected in larger N2 amplitudes for neutral trials. In the negative-evaluation condition, individuals hold a more negative or ambivalent view of their mobility experience and are more likely to experience self-threat and defensive emotional processing (Sherman and Cohen, 2006; Brockbank, 2024), so early attentional resources are more likely to be drawn into self-related and defensive processing rather than fine-grained discrimination of external painful versus neutral cues. In other words, when adopting a wealthy role with a positive evaluation of their mobility history, individuals can, from a relatively stable emotional state and with more economical use of attentional resources, make a clearer and more reliable early distinction between the poor target’s pain and neutral situations.

For the LPP component, pain stimuli elicited significantly larger amplitudes than neutral stimuli, irrespective of mobility direction or subjective evaluation. In contrast to the earlier N2 component, which mainly reflects more automatic perceptual processing, the LPP in this later time window is thought to reflect the stage after initial detection at which individuals, guided by task goals, actively maintain attention on the stimuli and engage in sustained cognitive evaluation, and it is widely regarded as a neural index of the late cognitive stage of empathy for pain (Cheng et al., 2012; Fan and Han, 2008). As participants performed pain judgment tasks, they needed to optimally allocate limited attentional resources to balance processing accuracy and cognitive demands (Kohlhas and Robertson, 2021). Compared with neutral stimuli that lack obvious threat, threatening pain cues are more likely to be subjected to sustained evaluative processing, and judgments about such painful events typically require greater cognitive resources and prolonged attention, consequently generating enhanced LPP amplitudes. Importantly, in the present study, neither mobility direction nor subjective evaluation modulated the LPP, whereas their influence emerged only at the N2 stage. This pattern suggests that class mobility experiences and positive appraisal mainly affect earlier, more automatic stages of empathic processing, while their impact at later stages that rely on higher-order cognitive control is relatively limited. This finding is consistent with stage-specific models of empathy (Decety and Jackson, 2006), which propose that individual traits or role-related differences are more likely to modulate early affective sharing, whereas later stages involving contextual understanding, meaning extraction, and inferential processing are driven primarily by the meaning of the situation and task demands, with a reduced contribution of individual differences. Thus, regardless of participants’ mobility experiences and subjective evaluations, strongly painful scenes elicited a robust and comparable LPP across conditions, because all observers engaged in relatively deep and convergent cognitive appraisal of the poor target’s pain.

Prior research conducted in individualistic cultural contexts has shown that upwardly mobile wealthy individuals often underestimate the difficulty of class mobility and exhibit lower empathy toward those who remain disadvantaged (Koo et al., 2023). In the present study, which was conducted within a collectivist cultural context, we observed a different pattern: even when participants merely simulated an upwardly mobile wealthy role, they still showed heightened early neural sensitivity to the pain of low-status individuals. One possible explanation for this discrepancy lies in cultural value orientations. Compared with individualistic cultures that emphasize personal goals and self-actualization, collectivist cultures place greater weight on collective goals, social harmony, and interpersonal obligations (Brewer and Chen, 2007; Yang et al., 2024). Interdependent self-construal suggests that in collectivist contexts, individuals tend to construe the self in interdependent terms, such that personal identity is closely embedded within social relationships, role obligations, and normative expectations (Markus and Kitayama, 1991). Individuals’ identity and behaviors are closely tied to their social groups, fostering greater attention to others’ needs and expectations, emotional sensitivity, and empathy (Duan et al., 2008; Klein et al., 2024). Moreover collectivist societies are often characterized by relatively low relational mobility, in which interpersonal ties are stable, social networks are more enduring, and reputational concerns carry greater weight (Yuki and Schug, 2012). Within such socioecological conditions, socioeconomically successful individuals may be more motivated to maintain relational credibility by displaying culturally valued, norm-consistent responses toward disadvantaged others. At the same time, there are also important methodological and measurement differences between the two lines of work. Koo et al. (2023) primarily used questionnaires and imagined scenarios to assess wealthy individuals’ self-reported empathy, attributions of responsibility for poverty, and support for redistribution policies–that is, explicit attitudes and policy-relevant judgments. By contrast, the present study employed ERP methods and a concrete pain-judgment task, focusing on the time course of neural responses to another person’s pain in a specific context. The former is closer to social evaluation and policy stance, whereas the latter captures rapid, partly automatic affective and cognitive processing, and these indices are not necessarily equivalent. Thus, the discrepancy between our findings and those of Koo et al. (2023) may reflect, on the one hand, the way collectivist cultural norms strengthen social connectedness and group belonging and thereby enhance sensitivity to others’ emotions at the neural level, and, on the other hand, differences in samples, task manipulations, and outcome measures. These possibilities should be examined more directly in future cross-cultural, multi-method research.

While considering the cultural and methodological differences discussed above, it is also important to situate the present findings within a broader theoretical framework of empathy. Existing evidence indicates that empathy is grounded in complex biological and neural systems and is jointly shaped by individual differences in genetics, age, sex, and cognitive resources (Luo et al., 2015; Hsu et al., 2023; Pua and Yu, 2024). At the same time, different cultural contexts modulate empathic responses at both neural and psychological levels (Cheon et al., 2011; Yi et al., 2024). Thus, the class-mobility- and culture-related effects observed in this study represent only one layer of a multi-level empathy system. Future research should adopt cross-cultural, multi-level designs that integrate genetic, cognitive, and cultural variables to more systematically examine how these factors jointly shape wealthy individuals’ empathic responses to the suffering of disadvantaged groups.

In summary, the present study used a virtual-society paradigm to examine how class mobility experiences and their subjective evaluation shape empathy for pain in a collectivist cultural context. The findings indicate that, within this simulated context, upward class mobility and positive appraisal of one’s mobility history primarily enhance wealthy-role participants’ early neural sensitivity to the pain of low-status individuals, whereas their influence on later stages of cognitive evaluation is relatively limited. These findings enrich our understanding of the relationship between social class mobility and empathy, particularly highlighting the distinct empathic mechanisms among the wealthy across cultural contexts. The present findings have practical implications for promoting social equity and philanthropy in collectivist societies. They suggest that initiatives targeting socioeconomically successful individuals may benefit from fostering reflective, socially embedded interpretations of upward mobility–for instance, encouraging “positive mobility narratives” that acknowledge collective support, social interconnectedness, and responsibility. Such narrative-focused approaches may be most effective when implemented alongside clear behavioral pathways (e.g., structured opportunities and normative supports) that facilitate sustained prosocial and equity-promoting engagement.

Nonetheless, several limitations should be noted. First, the findings are based on Chinese university students imagining wealthy roles in a virtual-society paradigm. Although manipulation checks confirming its effectiveness, the paradigm may still be susceptible to social desirability bias and reflects a relatively homogeneous middle-range family background. Therefore, the results may not directly generalize to wealthy individuals with actual class-mobility histories or to other cultural contexts. A further limitation is that role immersion was indexed only by manipulation checks rather than continuous ratings of identification or role-related emotions. Moreover, participants adopted four different wealthy roles across four weekly sessions in a within-subjects design; although the order of conditions was counterbalanced and procedures were kept consistent, repeated testing over multiple weeks may still introduce practice, fatigue, or variation in role immersion that cannot be fully ruled out. Second, low social status was only described in short vignettes, while the ERP stimuli were standardized limb-pain pictures without explicit class cues and involved a single poor target, so whether these ERP effects reflect empathy specifically toward “the poor” still needs to be tested in more ecologically valid paradigms with richer social information or interactive tasks. Finally, although the N2 effects were statistically significant, some of their absolute amplitudes were small, suggesting that larger samples and more sensitive, ecologically valid paradigms will be needed to robustly replicate and extend these findings. Future research could therefore recruit wealthy individuals with documented mobility experiences, adopt longitudinal designs, include cross-cultural comparisons, and combine ERP with behavioral and other multimodal measures in richer social contexts to build a more comprehensive picture of how class mobility shapes empathy for the poor.

## Data Availability

The raw data supporting the conclusions of this article will be made available by the authors, without undue reservation.
